# High Ascending Retrocecal Appendicitis in a Pediatric Patient Detected by Point-of-care Ultrasound

**DOI:** 10.5811/cpcem.2019.2.41682

**Published:** 2019-03-18

**Authors:** Takaaki Mori, Teng S. Shin, Gene Y.K. Ong

**Affiliations:** KK Women’s and Children’s Hospital, Department of Emergency Medicine, Singapore

## Abstract

A 10-year-old male presented to our pediatric emergency department with progressive, colicky abdominal pain for one day, associated with fever and non-bilious vomiting. He had a guarded abdomen with sluggish bowel sounds. He was noted to have poor perfusion with tachycardia, which resolved with fluid resuscitation. Abdominal radiograph demonstrated the presence of a circular radiopaque structure at the right hypochondrial region. Point-of-care ultrasound revealed an ascending appendicitis with signs of perforation, which was unusually located just at the inferior edge of the liver, over the right hypochondrium. The patient was immediately admitted to the surgical intermediate care unit. Urgent laparoscopic appendectomy was successfully performed, and the child was discharged well.

## INTRODUCTION

Abdominal pain in children is a common presentation to emergency departments (ED). The clinical approach to pediatric patients with abdominal pain in the ED requires a broad differential diagnosis. Pediatric patients with appendicitis often have a less-specific presentation, and perforation can occur rapidly.^1^ Appendicitis occurring in less-typical anatomical positions, especially ascending retrocecal appendicitis, can be difficult to diagnose.^2^ Missed or delayed diagnosis in perforated appendicitis can lead to serious morbidity and mortality.^3^ Pediatric appendicitis can present variably, but regardless of appendix location point-of-care ultrasonography (POCUS) can be useful.^4^

Published literature on the use of POCUS in the evaluation of pediatric abdominal pain and diagnosis of pediatric appendicitis is increasing.^4^ However, POCUS reports of ascending appendicitis in the pediatric population are uncommon. We present a case report of a pediatric patient with a high retrocecal appendicitis with perforation, which was detected by POCUS.

## CASE REPORT

**Image 1:**
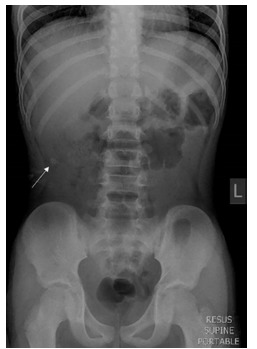
Abdominal radiography showing a circular radiopaque structure (arrow) over the right hypochondrium.

A previously healthy 10-year-old boy presented with an acute onset of periumbilical colicky abdominal pain of one day’s duration. It was rapidly progressive and became generalized. This was associated with tactile fever and two episodes of non-bilious vomiting. There was no associated diarrhea. The patient denied any recent sick contacts or overseas travel. At triage in the ED, he was febrile with a temperature of 38.2 degrees Celsius. He was tachycardic with a heart rate of 120 beats per minute and had a blood pressure of 90/61 millimeters (mm) of mercury. He had a respiratory rate of 20 breaths per minute and pulse oximetry was 99% on room air. His peripheral capillary refill time was delayed at three seconds. The triage nurses put him immediately in the resuscitation bay. Physical examination revealed an anicteric, lethargic child with a rigid abdomen and sluggish bowel sounds. Despite having generalized involuntary guarding of his abdomen, the child claimed the pain was “minimal.” However, he was noted to be wincing maximally when his abdomen was palpated over his right hypochondrium. He was clinically in shock and was promptly given fluid resuscitation with a rapid bolus of 20 milliliters per kilogram of normal saline. He responded well to the fluids with improved peripheral perfusion. An upright chest radiograph did not reveal any free air under the diaphragm. A supine abdominal radiograph showed a circular radiopaque structure suggestive of calcification (“stone”) over his right hypochondrium (Image 1).

**Image 2:**
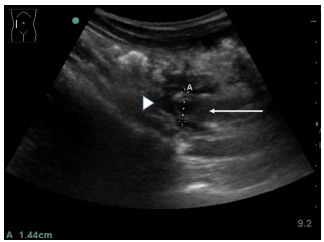
Point-of-care ultrasonography of the abdomen over the right hypochondrium demonstrated a dilated appendix (arrow) measuring 14.4 millimeters (A) with a surrounding rim of complex hypoechoic fluid (arrowhead), suggesting perforation.

POCUS was performed using Sonosite M-Turbo with a 2–5 megahertz curvilinear transducer. Visual cardiac contractility (parasternal long axis) showed a hyperkinetic left ventricle with good visual contractility. Initially, it was noted that his inferior vena cava was totally collapsed on spontaneous inspiration, which suggested that he was “volume depleted,” given his initial hemodynamic parameters. This improved post-fluid resuscitation. No gross abnormal hepatobiliary or renal abnormalities were noted. Unexpectedly, during the ultrasound examination of his right hypochondrium, a tubular structure measuring 14 mm in diameter with surrounding fluid at its end was noted just inferior to the liver’s surface (Image 2).

**Image 3:**
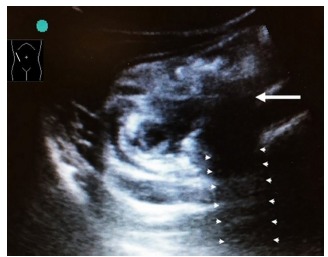
An object (arrow) within the dilated appendix with acoustic shadowing posteriorly (arrowheads) suggestive of an appendicolith.

The tubular structure could be traced caudally to the cecum, suggesting a high ascending retrocecal appendicitis. The complex fluid and loss of the submucosal echogenic layer surrounding the appendix suggested perforation. Further interrogation showed a structure with acoustic shadowing posteriorly suggestive of an appendicolith (Image 3) in the clinical context.

Ultrasound also revealed features of ileus. Based on these findings, he was admitted to the surgical intermediate care unit with a diagnosis of perforated appendicitis even before any hematological and biochemical laboratory investigation results were made available. After initiating intravenous antibiotics, the surgeons elected to obtain an urgent radiology abdominal ultrasound, which confirmed the findings of the initial POCUS. Soon after, he underwent an uneventful urgent laparoscopic appendectomy. Intraoperatively, surgical examination found that the cecum and appendix were unusually high, in close proximity to the liver’s inferior surface. The retrocecal appendix was noted to be inflamed with mid-shaft perforation. The base of the appendix was healthy. There was spillage of a fecolith into the paracolic gutter, with minimal fluid in the pelvis. Some adhesions were noted between the small bowel and abdominal wall. The rest of bowel, liver, and gallbladder were normal. Post-appendectomy, he was discharged well after a five-day hospitalization.

**CPC-EM Capsule**

What do we already know about this clinical entity?

*The clinical approach to unwell pediatric patients with abdominal pain requires a broad differential diagnosis. Pediatric appendicitis may be rapidly progressive and present atypically*.

What makes this presentation of disease reportable?

*There is a paucity of literature on the use of emergency department (ED) point-of-care ultrasound (POCUS) in the diagnosis of ascending appendicitis in the pediatric population*.

What is the major learning point?

*ED POCUS may provide critical clinical information and facilitate the evaluation and expedite management of atypically located, perforated appendicitis in children*.

How might this improve emergency medicine practice?

*Ascending appendicitis is notoriously difficult to diagnose, especially in children. ED POCUS should be considered as part of the clinical evaluation, especially in unwell pediatric patients*.

## DISCUSSION

This case report demonstrates the clinical utility of POCUS to detect and expedite the management of a rapidly progressive appendicitis with an unusual anatomical location, in a stoic child with a potentially misleading abdominal radiograph. His abdominal radiograph showed a “stone” over his right hypochondrium, which by location would usually indicate a gallbladder or renal stone. This “stone,” in retrospect, was a high appendicolith.

Unlike the ascending colon, the cecum and appendix are mobile and can vary in anatomical position. The appendix can be located in all directions in the pelvic cavity, with the most prevalent anatomical site of the appendix being at the descending peritoneal and retrocecal area.^5^ Therefore, presenting symptoms and signs of acute appendicitis depend greatly on the location of the appendix. Approximately half of the observational studies and case reports of ascending retrocecal appendicitis had atypical presentations with the pain located in either the right flank or hypochondrium, which contributed to initial misdiagnosis and delays in management in this time-sensitive condition.^6^ Most patients required the need for abdominal computed tomography (CT) for diagnosis.

Some authors advocate a right lower quadrant ultrasound for screening of pediatric appendicitis.^7^ While this is a “higher-yield” initial approach, a more systematic abdominal ultrasound surveillance is recommended if the appendix is not visualized during the initial screen. The findings of periappendiceal fluid or abscess, an intraluminal appendicolith, and loss of the submucosal echogenic layer of the appendix were associated with perforation.^7^–^9^ Prompt diagnosis of perforation is essential for optimal patient outcome. This case report illustrates the clinical value of POCUS to promptly diagnose and expedite the patient’s management despite the unusual clinical presentation.

While abdominal CT with contrast remains the classic gold standard to diagnose acute appendicitis, the principle of minimizing radiation exposure in the pediatric population should be strongly advocated.^10^ Ultrasound is an important imaging tool with high diagnostic sensitivity and specificity of 98% to detect appendicitis when performed by radiologists.^11^ With improved training for both adult and more recently pediatric emergency specialists, the clinical scope and utility of POCUS to diagnose and facilitate decision-making and management in pediatric appendicitis is also increasing.^4^,^12^ Benabbas et al., in a 2017 meta-analysis on the evaluation of pediatric appendicitis, reported ED POCUS to have a sensitivity of 86% and specificity of 91%.^4^

## CONCLUSION

This was an uncommon case of a perforated appendicitis in a child whose appendix was located just inferior to the liver. Physicians should be aware of atypical presentations of appendicitis in view of the variable anatomical positions of the appendix. POCUS can be a useful clinical adjunct to help differentiate surgical causes of abdominal pain, especially those with atypical presentations of appendicitis in the pediatric population.
